# GABA _A_ α4 is expressed in cerebrospinal fluid-contacting neurons and regulates swim behavior in developing zebrafish

**DOI:** 10.17912/micropub.biology.001882

**Published:** 2025-12-01

**Authors:** Wayne Barnaby, Sydney O'Malley, Gerald B. Downes

**Affiliations:** 1 Neuroscience and Behavior Graduate Program, University of Massachusetts Amherst, Amherst Center, Massachusetts, United States; 2 Biology Department, University of Massachusetts Amherst, Amherst Center, Massachusetts, United States

## Abstract

GABA _A_ receptors are present in hindbrain and spinal cord networks, playing a pivotal role in regulating locomotion. In this study, we demonstrate that mutations in the *gabra4* gene, which encodes the α4 subunit of GABA _A_ receptors, result in increased swim velocity of larval zebrafish. We also show that this gene is selectively expressed within spinal cord cerebrospinal fluid contacting neurons (CSF-cNs). Given the significance of these neurons in modulating locomotion, our findings support a model in which compromised α4 function leads to an increase in CSF-cN activity, causing a subtle, hyperactive swimming phenotype.

**Figure 1. gabra4 mutant behavior and transcript expression in 48 hpf zebrafish:**
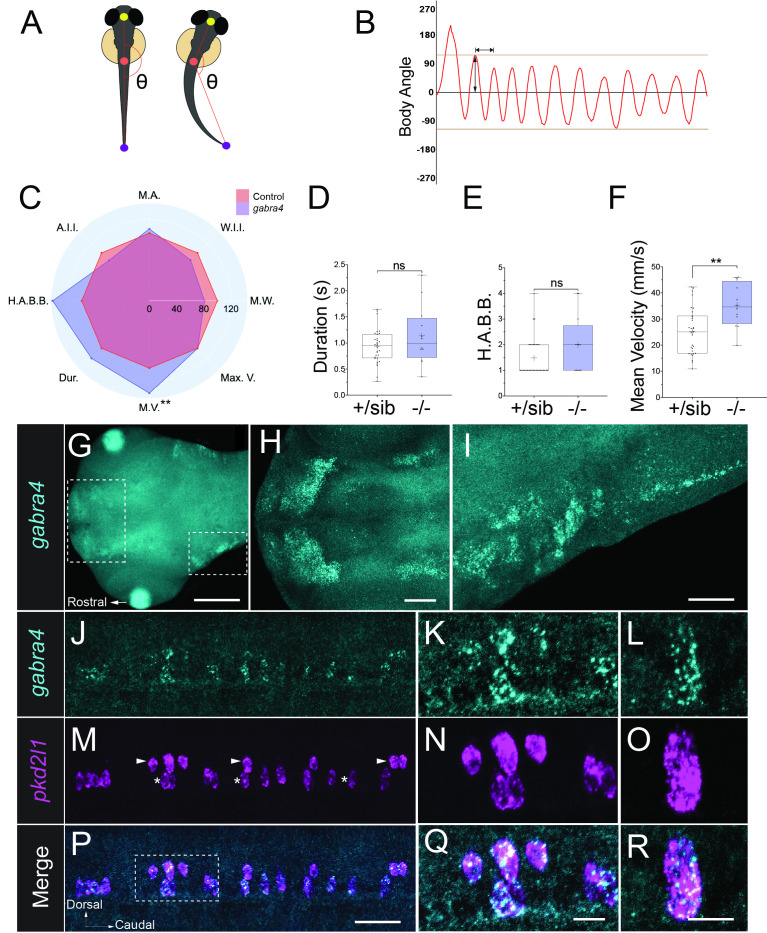
**(A)** Schematic, modified from Teicher *et al.* 2025, that illustrates the landmarks used to generate body angles. **(B)** Schematic of the body angles produced over time during a normal escape response. Beige lines represent the 110º and -110º thresholds for a high amplitude body bend (H.A.B.B.) or C bend. The horizontal line with arrow heads shows the measurement of period length. The vertical line with arrow heads represents body bend amplitude. **(C)** Radar plot of *gabra4* mutant kinematic behavior averages normalized to sibling controls. Average Control values are 100%. M.A., Mean Amplitude; W.I.I., Wavelength Irregularity Index; M.W., Mean Wavelength; Max V., Maximum Velocity; M.V., Mean Velocity; Dur., Duration; H.A.B.B., High Amplitude Body Bends; A.I.I., Amplitude Irregularity. Only Mean Velocity is significantly different compared to controls. **P<0.01 using unpaired Welch’s *t* -tests. **(D-F)** Box plots of averages with standard deviation for *gabra4* mutants and sibling controls. **(D)** No significant differences were observed in swimming duration, shown in milliseconds or **(E)** H.A.B.B. per response. **(F)** However, *gabra4* mutants exhibit an increase in Mean Velocity in millimeters per second. **(G-R)** Confocal analysis of *gabra4* and *pkd2l1* expression. **(G)** Shows expression of *gabra4* in the developing brain. The regions outlined by dashed the white boxes are shown at higher magnification in **H** and **I** . **(J)** *gabra4* is expressed in a subset of cells in the spinal cord. The region outlined by the dashed box in P is shown at higher magnification in **K** and **L** . **(M-O)** *pkd2l1* is expressed in spinal cord CSF-cNs. CSF-cNs consist of two distinct subtypes located around the central canal, a dorsal population ( *arrowheads* ) and a ventral population ( *asterisks* ). **(P-R)** Merged channels show extensive colocalization of *gabra4* and *pkd2l1* , indicating that *gabra4* is expressed in dorsal and ventral subtypes of CSF-cNs. Scale bars indicate 50μm, 10μm, and 5μm.

## Description

GABA _A_ receptors mediate rapid responses to the neurotransmitter GABA. Typically inhibitory, these receptors play key roles in regulating vertebrate locomotor behavior. GABA _A_ receptors are composed of heteropentamers drawn from a pool of at least 19 different subunits. Each subunit is encoded by a distinct gene, with its own spatial and temporal pattern of expression (Chua & Chebib, 2017; Sieghart & Sperk, 2002; Simon et al., 2004). The distinct expression patterns of individual subunits suggest their involvement in specific locomotor circuits. Investigating the expression patterns of GABA _A_ receptor subunits can provide insights into the organization and function of these circuits.

Developing zebrafish are a leading system to analyze the cellular and molecular mechanisms that mediate locomotion. There are 22 identified zebrafish GABA _A_ receptor subunits, and several pharmacological and genetic studies have demonstrated that blocking or mutating these subunits leads to hyperactive swimming behavior (Baraban et al., 2005; Liao et al., 2019; B. D. Monesson-Olson et al., 2018; Sadamitsu et al., 2021; Samarut et al., 2018). In a previous study, we focused on α subunits responsible for controlling zebrafish larvae swimming behavior, which revealed that mutations in *gabra3* (encoding α3), *gabra4* (encoding α4), and *gabra5* (encoding α5) alone or in combination with one another generated hyperactive swimming behavior (Barnaby et al., 2022). We observed that loss-of-function mutations in α3 combined with mutations in α4 caused an increase in High Amplitude Body Bends (H.A.B.B.) at 48 hours post-fertilization (hpf). Meanwhile, mutations in α5 combined with mutations in α4 resulted in increased swimming duration at the same time point. However, this earlier study limited its analysis to only two swimming parameters, H.A.B.B. and swimming duration, and cell-type specific expression was not explored.

In this study, we expand our analysis of *gabra4* mutants by examining six additional kinematic parameters in response to touch stimuli. Touch-evoked responses were recorded using a high-speed video camera and, using the Marigold web app developed by our lab, kinematic analysis was performed (Teicher et al., 2025). We found that most parameters remained indistinguishable from sibling controls (Figures A-F), with the exception of mean velocity, where *gabra4* mutant escape responses exhibited significantly higher velocity (Figure F; 35.22 mm/s for α4 mutants compared to 25.27 mm/s for sibling controls). These results indicate that mutations in α4 cause a subtle yet significant hyperactive swimming phenotype.

To investigate the cellular mechanisms through which α4 regulates escape behavior, we next performed expression analysis using *in situ* hybridization chain reaction on 48 hpf larvae. In our previous work, we observed *gabra4* expression in the lateral hindbrain and a population of ventral spinal cord cells along the central canal (B. Monesson-Olson et al., 2018). Given the distribution of these cells, we proposed that these cells are Kolmer-Agduhr cells or cerebrospinal fluid contacting neurons (CSF-cNs), which line the ventricles and central canal of the spinal cord. Confirming our earlier results, here we observed that *gabra4* is expressed in a subset of cells in the forebrain and lateral hindbrain (Figure G-I), and an array of ventrally located cells in the spinal cord (Figure J-L). *Polycystic kidney disease 2-like 1* ( *pkd2l1* ), also known as TRPP3, is a non-selective cation channel that serves as a marker for spinal cord CSF-cNs (Djenoune et al., 2014). There are two distinct subtypes of CSF-cNs, dorsal and ventral populations (Djenoune et al., 2017). We found that *gabra4* is extensively coexpressed with *pkd2l1* in both dorsal and ventral subtypes of CSF-cNs in the spinal cord (Figure M-R). These data suggest that the hyperactive phenotype observed in *gabra4* mutants could be due to abnormal CSF-cN function.

CSF-cNs respond to spinal cord movement and provide a means to regulate zebrafish locomotion. Bending of the tail, during escape responses or swimming, activates GABAergic CSF-cNs, which then inhibit neurons in the spinal cord and hindbrain to control posture and the vigor of body flexions (Muñoz-Montecinos et al., 2022; Wyart et al., 2023). Impaired CSF-cN function results in lower high-amplitude body bends, slower swimming velocities, and deficient postural control (Böhm et al., 2016). Although we have not ruled out that impaired α4 expression in supraspinal cells generates hyperactive behavior, given the known roles of CSF-cNs in controlling locomotion, we propose that loss of α4 enhances the activity in at least a subset of these cells to cause increased swimming velocities.

Based upon the subcellular distribution of α4 in mammalian systems, α4 could regulate CSF-cNs through synaptic and/or extrasynaptic mechanisms (Bohnsack et al., 2016; Liang et al., 2006). Extrasynaptic GABA _A_ receptors sense ambient concentrations of GABA (Belelli et al., 2009). Given that cerebrospinal fluid typically contains GABA and that spinal cord CSF-cNs are exposed to cerebrospinal fluid in the central canal, α4-containing GABA _A_ receptors may provide tonic inhibition to tune CSF-cN regulation of escape and locomotion. α4-containing GABA _A_ receptors can also be found within synapses (Bohnsack et al., 2016; Liang et al., 2006). Although it is not clear what neurons innervate zebrafish CSF-cNs, ultrastructural analysis reveals that they do contain inhibitory synapses (Djenoune et al., 2017). In mice, CSF-cNs have been shown to innervate other CSF-cNs to form recurrent connections (Nakamura et al., 2023). It is possible that α4 mediates similar connectivity in zebrafish.

## Methods

*Zebrafish Maintenance and Breeding*

Adult zebrafish were maintained according to standard procedures, with the zebrafish facility on a 14-hour light/10-hour dark cycle. Embryos and larvae were kept at 28.5℃ in E3 media and staged according to morphological criteria (Kimmel et al., 1995; Parichy et al., 2009). All animal procedures for this study were approved by the University of Massachusetts Amherst Institutional Animal Care and Use Committees (IACUC) under assurance number 3551-1 with the Office of Laboratory Animal Welfare.

α *4 Mutant Line Generation and Genotyping*

The generation of the α4 mutant line and the primers used for CRISPR-STAT analysis were described in our previous study (Barnaby et al., 2022). The *umz504* mutant allele contains an 11 bp deletion in *gabra4* . To genotype gabra4 mutant larvae, fish were euthanized by overdose with MS-222 (pH 7.0; Sigma-Aldrich, St. Louis, MO, USA) the genomic DNA was extracted using the Extract-N-Amp Tissue PCR Kit (Sigma-Aldrich) following the manufacturer’s instructions. A region of exon 2 encompassing the mutation was amplified by PCR using the forward primer 5’-GCTTCAGTTTGCTCTGTGTTGT-3’ and the reverse primer 5’-CACTTAGTAAACAGCGTGCGAC-3’. PCR was performed with AmpliTaq Gold DNA Polymerase (Applied Biosystems/Thermo Fisher Scientific, Waltham, MA, USA) using an annealing temperature of 59 °C. Products were resolved by agarose gel electrophoresis, with wild-type and mutant alleles producing 267-bp and 256-bp fragments, respectively.

*Behavioral Analysis*

Touch stimuli was applied to the head of 48 hpf larvae using a 3.22/0.16g of force von Frey filament and swimming responses were recorded using a high-speed digital camera (XStream 1024, IDT vision) as previously described (Barnaby et al., 2022; Friedrich et al., 2012; McKeown et al., 2012). Following video recordings, the larvae were genotyped as described above. Videos were uploaded and analyzed using the Marigold (Teicher et al., 2025). To determine significant differences, the following statistical tests and software were used. Welch’s *t* -test and Ordinary 1-way ANOVA were used as indicated. When *t* -tests were applied, *F* tests were used to compare variance. When ANOVAs were applied, multiple comparison tests were used where test groups were compared against wild-type controls. A Dunnett test was used to correct against familywise errors. Statistical tests were performed and plots and figures were generated using Prism (GraphPad Software).

*In Situ Hybridization Chain Reaction*

*In situ* hybridization chain reactions were performed using standard methods (Choi et al., 2018). In summary, whole mount samples were dehydrated and rehydrated with MeOH and PBST respectively. Probe solutions were made using customized oligo pools (IDT) diluted in a hybridization buffer (Molecular Instruments). All 37℃ incubations were performed in a temperature controlled water bath. Amplification was carried out by using hairpins conjugated to a B1, B2, or B3 initiator diluted in an amplification buffer (Molecular Instruments). Samples were mounted in Vectashield mounting media and imaged using a Zeiss LSM710 confocal microscope equipped with 40x and 63x oil immersion objectives. Images were captured using ZenBlack software and employed 488, 555 and 639 nm laser lines. Images were processed using Fiji (Schindelin et al., 2012) and Adobe Illustrator software (Adobe Systems, Mountain View, CA).
